# Hyperacute versus Subacute Coiling of Aneurysmal Subarachnoid Hemorrhage a Short-term Outcome and Single-Center Experience, Pilot Study

**DOI:** 10.3389/fneur.2016.00079

**Published:** 2016-06-16

**Authors:** Abdulrahman Mostafa Ibrahim Ali, Ghada Abdel Hady Ossman Ashmawy, Ayman Youssef Ezeddin Eassa, Osama Yassin Mansour

**Affiliations:** ^1^Department of Neurology and Psychiatry, Faculty of Medicine, University of Alexandria, Alexandria, Egypt; ^2^Stroke Center, Alexandria University Hospital, Alexandria, Egypt

**Keywords:** subarachnoid hemorrhage, endovascular treatment, aneurysm, timing, outcome

## Abstract

**Background:**

After the initial subarachnoid hemorrhage (SAH), rebleeding is the major cause of morbidity and poor outcome, which is maximal in the first 24 h. We supposed that the coiling of ruptured intracranial aneurysms within 24 h of SAH is related to the improved clinical outcome compared with coiling 24 h after aneurysmal SAH.

**Objective:**

We examined whether coiling ruptured aneurysms within 24 h of aneurysmal SAH is associated with better early 24 h and late 30 days outcome.

**Method:**

This prospective study was carried on 30 patients with aneurysmal SAH presenting to the Alexandria University Hospital and Insurance Main Hospital during the period from February 2013 to May 2014. They were divided into two groups: Group I (10 patients treated within 24 h of presentation) and Group II (20 patients treated after 24 h of presentation). Time interval from presentation to treatment were 78.60 and 10.60 h for subacute and hyperacute group, respectively. All patients were treated with endovascular coiling. Change between admission and immediate postoperative World Federation of Neurosurgeons classification Scale was measured for early outcome, while remote outcome was measured by modified Rankin Scale at 30 days follow-up.

**Results:**

There was a clinical improvement regarding early 24 h outcome (weighted by postoperative WFNS grade) and on late 30 days outcome (weighted by Modified Rankin Scale Score) in the group managed within 24 h versus who were treated after 24 h (*P* = 0.049 and *P* = 0.024, respectively). There was a significant decrease in the incidence of clinical rebleeding detected by postoperative computed tomography (CT) of the brain in patients undergoing aneurysm treatment within 24 h (*P* = 0.029).

**Conclusion:**

The study affirms evidence that the management of ruptured intracranial aneurysms within 24 h of SAH is associated with better immediate and short-term clinical outcome.

## Introduction

Aneurysmal subarachnoid hemorrhage (SAH) is a subset of stroke that occurs at a relatively young age (median 55 years) and has a high rate of morbidity (25%) and case fatality (35%). In SAH patients who survive the first few days after bleeding, rebleeding is an important contributor to poor outcome (1).

For the last two decades, there has been proof that most rebleeds occur during the first 24 h after SAH. Multiple studies have proven that the incidence of rebleeding is maximal during the first 24 h after SAH with rates of 4.1–17.3% reported (2). Most studies reporting outcomes associated with rebleeding within 24 h have demonstrated case-fatality rates approaching 65%, some as high as 80%, and incidences of 10% (3).

The time interval from presentation to management of acutely ruptured aneurysms (endovascular coiling) has been the issue of considerable debate (2). Earlier treatment of the ruptured intracranial aneurysm is associated with the lower incidence of rebleeding but was historically considered as a higher risk than a delayed management (4).

Few studies analyzed the management of ruptured aneurysms within 24 h of SAH, most of them were in retrospective manner not prospective one like our study (5). Also, most of them were comparing any intervention (both surgery and endovascular coiling) in addition to defining early treatment at the first 72 h after SAH, unlike our study. To date, not enough published data are present that compare hyperacute treatment (within 24 h of aneurysmal SAH) with treatment at more than 24 h post-SAH (6). We believe that our work is somewhat novel.

We supposed that coiling of ruptured intracranial aneurysms within 24 h of SAH is related to improved clinical outcome compared with coiling 24 h after aneurysmal SAH.

## Materials and Methods

### Subject

This study was carried out among patients with SAH presenting to the Alexandria University Hospital and Insurance Main Hospital during the period from February 2013 to May 2014. The study consisted of 30 patients with aneurysmal SAH divided into two groups: Group I: study group (10 patients); hyperacute aneurysmal SAH referred for coiling and treated within 24 h from presentation and Group II: study group (20 patients); subacute aneurysmal SAH referred for coiling and treated after 24 h from presentation. All patients underwent clinical evaluation that include personal data, medical data, past medical history, social history, and complete clinical neurological examination. Early 24 h outcome was measured by World Federation of Neurosurgeons classification Scale (7) at 24 h postoperatively, and late 30 days outcome was measured by the modified Rankin Scale at 30 days follow-up by contacting the participants by telephone and arranging an interview (8). Rebleeding was detected by an increase of Fisher grade through evidence of blood in the subarachnoid space, ventricular system, or brain parenchyma by follow-up computed tomography (CT). Rebleeding may be symptomatic [associated with acute clinical deterioration (decrease of one or more grade on Glasgow Coma Score) and confirmed by repetitive CT scan] or asymptomatic [confirmed by repetitive CT scan only]. Pre endovascular evaluation included CT of the brain, CT angiography, and initial diagnostic subtraction angiography (DSA). All the ethical committee data and request are included, including the consent from all patients.

### Procedure

The endovascular treatment was started by puncture of the femoral artery, followed by introducing the guide-catheter (6F) to be positioned in the cervical vessel that allows access to the aneurysm. Angiographic assessment by a rotational angiographic data set to obtain 3D reconstruction of the aneurysm. We started with possible largest coil then smaller one and so on, under fluoroscopic roadmap guidance, to serves framing the theoretical boundary of the aneurysm (framing coil). Appropriately sized smaller coils were subsequently delivered to fill the aneurysm fundus, anticoagulation with intravenous heparin, or heparinization during treatment was employed. In some cases, we used simple coiling while in other cases where simple coiling is impossible, we used adjunctive endovascular maneuvers (assisted coiling) that involve the one of the following:

- Remodeling of the neck (neck selective technique), in case of wide neck, such as:
  - Balloon-assisted coiling
  - Stent-assisted coiling
- Remodeling of the sac (branch selective technique), such as catheter-assisted catheter technique.

Endovascular treatment to all patients and validation of data in terms of angiographic findings and treatment efficacy were done by consultant neuro-interventionist, Osama Yassin Mansour. Outcome assessment was done by the corresponding author and reviewed by the other two authors.

### Postoperative Management

The treatment of vasospasm was managed by Triple-H therapy (induced hypertension, hypervolemia, and hemodilution) and endoluminal angioplasty. Post-endovascular evaluation included postoperative CT of the brain to exclude any postoperative complications (intra-cerebral and/or intra-ventricular hemorrhage, brain oedema, or cerebral infarction).

Our policy is to manage all cases within 24 h of aneurysmal SAH. Management delays were due to the transfer from other hospitals or from rural areas, hospital logistical delays (access to operating rooms and nursing staff), and delayed diagnoses.

The study protocol was reviewed and approved by data safety monitoring board and subsequently by the local institutional review board. Each patient or his relative signed a written informed consent before the procedure. Relevant data were recorded on a standard case reporting form.

Data were fed to the computer and analyzed using IBM SPSS software package version 20.0. The distributions of quantitative variables were tested for normality using Kolmogorov–Shapiro–Wilk test and D’Agstino test, also Histogram and QQ plot were used for vision test. For normally distributed data, comparison between two independent populations was done using independent *t*-test. Significance of the obtained results was judged at the 5% level.

## Results

Statistical analysis demonstrated no significant difference in age, gender, hypertension, diabetes mellitus, admission grade, size, and location between those managed within 24 h (hyperacute) and those managed after 24 h (subacute). We had only one patient who died during the follow-up in the subacute group (Table 1).

**Table 1:** **Comparison between the two studied groups as regards demographic data and risk factors**.

	Subacute group (after 24 h, 20 patients)	Hyperacute group (within 24 h, 10 patients)	*P* value
Age	50.65 ± 12.40	50.50 ± 15.81	0.977
Female	12 (60)	3 (30)	0.121
HTN	13 (65)	7 (70)	1.000
DM	4 (20)	3 (30)	0.657
WFNS grade (admission grade)			0.313
1	9 (45)	2 (20)	
2	5 (25)	4 (40)	
3	3 (15)	4 (40)	
4	2 (10)	0 (0)	
5	1 (5)	0 (0)	
Mortality	1 (5)	0 (0)	
Size (mm)			1.000
Less than 7	11 (55)	6 (60)	
7–12	8 (40)	4 (40)	
13–24	1 (5)	0 (0)	
More than 25	0 (0)	0 (0)	
Location			1.000
Anterior circulation	14 (70)	7 (70)	
ICA	3 (15)	1 (10)	
ACOM	6 (30)	5 (50)	
ACA	1 (5)	1 (10)	
MCA	4 (20)	0 (0)	
Posterior circulation	6 (30)	3 (30)	
BA	4 (20)	1 (10)	
PCOM	1 (5)	1 (10)	
PCA	0 (0)	1 (10)	
PICA	1 (5)	0 (0)	

Early 24 h outcome was measured by clinical improvement weighted by reduction in WFNS grade (≥1 grade). In the hyperacute group,; there were eight patients (80%) who were clinically improved, compared to six patients (30%) in the subacute group with statistical significant difference between both groups (*P* = 0.019) (Table 2).

**Table 2:** **Comparison between the two studied groups as regards outcome**.

	Subacute group (after 24 h, 20 patients)	Hyperacute group (within 24 h, 10 patients)	*P* value
Early 24 h outcome			0.019*
Better outcome	6 (30)	8 (80)	
Worse outcome or no change	14 (70)	2 (20)	
Late 30 days outcome			0.024*
Good outcome	9 (45)	9 (90)	
High morbidity	10 (50)	1 (10)	

*P value ≤0.05.

Dichotomization of the mRS outcome data into good outcome (mRS 0–2) and high morbidity (mRS 3–5) also demonstrated a statistical significant difference. The patients were followed up for a mean of 31 days (range between 26 and 34 days). Also, 90% (*n* = 9) of cases in hyperacute group achieved a good outcome (mRS 0–2) on late 30 days follow-up, while only 45% (*n* = 9) of cases of subacute group achieved a good outcome with statistical significant difference between both groups (*P* = 0.024) (Table 2).

Rebleeding was present in 40% (*n* = 8) of cases in subacute group only. Statistically significant decrease in incidence in clinical rebleeding detected by postoperative CT of the brain was observed in patients undergoing aneurysm treatment within 24 h (0 versus 40%, *P* = 0.029) (Table 3).

**Table 3:** **Comparison between the two studied groups as regards to the incidence of rebleeding**.

	Subacute group (after 24 h, 10 patients)	Hyperacute group (within 24 h, 10 patients)	*P* value
Symptomatic rebleeding	8 (40)	0 (0)	0.029*

*P ≤ 0.05.

Time interval from presentation to treatment was significantly higher in subacute group compared to hyperacute group (*P* = 0.0001), wherein the subacute group, the mean time interval from presentation to treatment was 78.60 versus 10.0 h in the hyperacute group (Table 4).

**Table 4:** **Comparison between the two studied groups as regards to time interval from presentation to treatment**.

	Subacute group (after 24 h, 10 patients)	Hyperacute group (within 24 h, 10 patients)	*t*	*P* value
Time interval from presentation to treatment (hours)
Min–max	36.0–130.0	32.0–110.0
Mean ± SD	78.60 ± 25.057	10.60 ± 3.373	11.923	0.0001*
Median	80.0	10.0		

*P ≤ 0.05.

**t*: Student’s *t*-test*.

## Discussion

The time interval from presentation to management of acutely ruptured aneurysms (endovascular coiling) has been the issue of considerable debate (2). Definitions have been developed for specific time intervals that have been considered better or worse options during the last half century. Late treatment usually points to 10 days post-SAH; intermediate treatment points to days 4–10; early treatment points to the first 3 days; and ultra-early treatment points to surgery during the first 24 h. We point to all treatment after the first 24 h as delayed, a group that included the previously mentioned early, intermediate, and late treatment (9).

After the initial SAH, rebleeding is the major cause of morbidity and poor outcome (10, 11). Among aneurysms that rebleed, approximately 20% do so in the first 2 weeks, 30% by the end of the first month, and 40% by the end of 6 months. Beyond 6 months, rerupture occurs at a rate of approximately 3% per year (12). No infallible rules predict which patients will have recurrent hemorrhages (13). Most studies reporting outcomes associated with rebleeding within the first 24 h have demonstrated case-fatality rates approaching 65%, some as high as 80%, and incidences of 10% (3).

Securing the ruptured intracranial aneurysm within the first 24 h after SAH has showed reduction in the risk of rebleeding. Earlier coiling of the ruptured aneurysm also means that medical or endovascular management for vasospasm/delayed ischemic neurological deficits can be initiated instantly (14).

In our cohort, 80% (*n* = 8) of cases of hyperacute group achieved a better outcome on early 24 h follow-up compared to 30% (*n* = 6) of cases of subacute group with statistical significant difference between both groups (*P* = 0.019). Our findings were in consistence with a retrospective 11-year single-center study done by Phillips et al., who suggested that treatment within 24 h was related to a better neurological outcome (15). Our findings are also in consistence with Bergui and Bradac, who reviewed the outcome of 45 consecutive patients treated acutely by coiling after SAH and found that about half had a favorable outcome; a promising result in accordance with some surgical and endovascular reports proposing early aggressive treatment of an aneurysm causing SAH (16). These findings are also in agreement with a study done by Wong et al., who reported that treatment within 24 h is associated with better clinical outcomes of aneurysmal SAH, as less clinical rebleeding happens. The same study reported that aneurysm treatment carried out within the 24 h may improve clinical outcome and halve the clinical rebleeding in poor-grade SAH patients (17).

In the present cohort, 90% (*n* = 9) of cases in hyperacute group achieved a good outcome (mRS 0–2) on late 30 days follow-up, while only 45% (*n* = 9) of cases in subacute group achieved a good outcome with statistical significant difference between both groups (*P* = 0.024). The mortality in our patients (5%, *n* = 1) was more or less similar to that of the patients in the ISAT study (8.1%), this can be partially explained by the fact that the higher prevalence of patients with milder clinical presentation, WFNS grades (1 and 2), in both cohorts (70% of patients in our study and 88% of patients in the ISAT study) (18).

Regarding low prevalence of grades 4 and 5 (0%, *n* = 1) in group of patients treated within 24 h, which was due to small number enrolled during study period, we think that it is a good chance that shows the true real practice, where those higher grade patients with higher possibility of having ICH or IVH are preferred to be managed with open surgery to additionally evacuate ICH or to deploy shunting for CSF.

In our cohort, symptomatic rebleeding was present in 40% (*n* = 8) of cases in subacute group only, all of the rebleeding cases had hypertension while six out of eight had poor admission grade (WFNS grade 3 or 4), which can explain the occurrence of rebleeding among these cases. Statistically significant decrease in incidence of clinical rebleeding was observed in patients undergoing aneurysm treatment within 24 h (0 versus 40%, *P* = 0.029). These findings could be explained by significant longer door to puncture time interval in subacute group when compared to hyperacute group (*p* = 0.0001). In 2001, Ohkuma et al. (19) examined 273 patients who were admitted to their institution within 24 h after the initial SAH bleeding and recorded that 13.6% of patients with SAH suffered rebleeding in the ambulance or before admission to the referring hospital, which was related to systolic arterial pressure >160 mmHg. Our result went also in agreement with Guo et al., who showed that rebleeding found in 24 patients (34.3%) within 3 h compared to 44 patients (62.9%) within 6 h with aneurysm size and systolic arterial pressure were the independent risk factors for aneurysmal rebleeding (20). In other study, Tanno et al. reported that rebleeding occurs more frequently in the earlier period after the initial SAH than previously believed and found that rebleeding occurred in 65 patients (35.9%) within 3 h and 88 patients (48.6%) within 6 h after the initial SAH, which was not related to hypertension, as systolic arterial blood pressure prior to rebleeding was most common between 120 and 140 mmHg (4).

The primary limitation of this study is the way of enrollment of our cases, which was based on availability of cases and not on randomization. The present study was also limited by the small sample size that may be due to the narrow time frame of the study. This may shed light on the real practice behavior of referring such patients at least in our region that needs to be changed, so further study with larger sample size is required to confirm the findings.

## Conclusion

Aneurysm treatment within 24 h may be associated with better clinical outcome. The study affirms evidence that management of ruptured intracranial aneurysms within 24 h of SAH is associated with better immediate and short-term clinical outcome.

## Author Contributions

All authors listed have made substantial, direct, and intellectual contribution to the work and approved it for publication.

## Conflict of Interest Statement

The authors declare that the research was conducted in the absence of any commercial or financial relationships that could be construed as a potential conflict of interest.

## Abbreviations

SAH, subarachnoid hemorrhage; WFNS, World Federation of Neurosurgeons classification Scale.
